# Cord blood adipokines, neonatal anthropometrics and postnatal growth in offspring of Hispanic and Native American women with diabetes mellitus

**DOI:** 10.1186/s12958-015-0061-9

**Published:** 2015-06-26

**Authors:** April M. Teague, David A. Fields, Christopher E. Aston, Kevin R. Short, Timothy J. Lyons, Steven D. Chernausek

**Affiliations:** Department of Pediatrics CMRI Metabolic Research Program, University of Oklahoma Health Sciences Center, 1200 Children’s Ave, Suite 4500, Oklahoma City, OK 73104 USA; Section of Endocrinology and Diabetes, Department of Internal Medicine, University of Oklahoma Health Sciences Center, 1000 N Lincoln Blvd, Suite 2900, Oklahoma City, OK 73104 USA; Harold Hamm Diabetes Center, University of Oklahoma Health Sciences Center, 1000 N Lincoln Blvd, Suite 1200, Oklahoma City, OK 73104 USA; Centre for Experimental Medicine, Queen’s University Belfast, Grosvenor Road, Belfast, BT12 6BA Northern Ireland UK

**Keywords:** Prenatal programming, Maternal diabetes, Fetal adipokines

## Abstract

**Background:**

Offspring of women with diabetes mellitus (DM) during pregnancy have a risk of developing metabolic disease in adulthood greater than that conferred by genetics alone. The mechanisms responsible are unknown, but likely involve fetal exposure to the *in utero* milieu, including glucose and circulating adipokines. The purpose of this study was to assess the impact of maternal DM on fetal adipokines and anthropometry in infants of Hispanic and Native American women.

**Methods:**

We conducted a prospective study of offspring of mothers with normoglycemia (Con-O; *n* = 79) or type 2 or gestational DM (DM-O; *n* = 45) pregnancies. Infant anthropometrics were measured at birth and 1-month of age. Cord leptin, high-molecular-weight adiponectin (HMWA), pigment epithelium-derived factor (PEDF) and C-peptide were measured by ELISA. Differences between groups were assessed using the Generalized Linear Model framework. Correlations were calculated as standardized regression coefficients and adjusted for significant covariates.

**Results:**

DM-O were heavier at birth than Con-O (3.7 ± 0.6 vs. 3.4 ± 0.4 kg, *p* = 0.024), but sum of skinfolds (SSF) were not different. At 1-month, there was no difference in weight, SSF or % body fat or postnatal growth between groups. Leptin was higher in DM-O (20.1 ± 14.9 vs. 9.5 ± 9.9 ng/ml in Con-O, p < 0.0001). Leptin was positively associated with birth weight (*p* = 0.0007) and SSF (*p* = 0.002) in Con-O and with maternal hemoglobin A1c in both groups (Con-O, *p* = 0.023; DM-O, *p* = 0.006). PEDF was positively associated with birth weight in all infants (*p* = 0.004). Leptin was positively associated with PEDF in both groups, with a stronger correlation in DM-O (*p* = 0.009). At 1-month, HMWA was positively associated with body weight (*p* = 0.004), SSF (*p* = 0.025) and % body fat (*p* = 0.004) across the cohort.

**Conclusions:**

Maternal DM results in fetal hyperleptinemia independent of adiposity. HMWA appears to influence postnatal growth. Thus, *in utero* exposure to DM imparts hormonal differences on infants even without aberrant growth.

## Background

Children born to mothers with diabetes mellitus (DM) are at increased risk for development of type 2 diabetes mellitus (T2DM) and other metabolic abnormalities later in life. The mechanisms of transmission to offspring are not yet understood and go beyond straightforward Mendelian inheritance of susceptibility genes. Several lines of evidence implicate the *in utero* environment as an important factor. First, the risk associated with maternal DM is greater than that of paternal DM. Second, fetal exposure to the maternal diabetic milieu confers additional risk, independent of other factors. Dabelea and colleagues reported that offspring conceived after their mother’s DM diagnosis are nearly four times more likely to develop T2DM than their siblings born before their mother developed diabetes, supporting a key role for the fetal environment [1].

The *in utero* transmission of risk for diabetes from a mother to her offspring, whether genetic or environmental, potentially involves modulation of circulating cytokines that influence appetite regulation, insulin action, or other metabolic functions. We selected leptin and high-molecular-weight adiponectin (HMWA) for evaluation because both are produced by adipose tissue and have been shown in studies of adults or experimental animals to be associated with insulin resistance, adiposity, fat metabolism, and inflammation.

Pigment epithelial-derived factor (PEDF) is a serpin-like protein lacking inhibitory function that was selected for study because expression is known to be influenced by diabetes and obesity [2, 3], circulating levels are related to vascular complications of diabetes and insulin sensitivity [4–6], and it is produced by adipose tissue, liver, and placenta [7–9]. One prior report found PEDF expression decreased in placentae from stillborn [10] and a pilot study noted increased cord blood levels in pre-eclampsia [11].

We investigated the impact of maternal DM on the concentrations of these adipokines in fetal circulation (the *in utero* milieu) and anthropometric measures at birth and at 1-month of age in offspring of Hispanic and Native American women, a population at intrinsically increased risk for the development of T2DM [12].

## Methods

Participants were recruited from the University of Oklahoma Medical Center (Oklahoma City, OK), the Chickasaw Nation Medical Center (Ada, OK) and the Choctaw Nation Health Care Center (Talihina, OK) between April 2010 and October 2013. Signed informed consent was obtained from a parent of each infant in accordance with the University of Oklahoma Health Sciences Center, Chickasaw Nation and/or Choctaw Nation of Oklahoma Institutional Review Boards, each of which approved the study.

Offspring of self-identified Native American and Hispanic women with diagnosed gestational or pre-existing type 2 diabetes mellitus (DM-O group) or confirmed normoglycemic pregnancies (Con-O group) were enrolled at birth into a prospective, longitudinal study of the impact of *in utero* exposure to DM on postnatal growth. Maternal gestational and type 2 diabetes were diagnosed according to ADA guidelines [13]. Infants with major malformations, CNS deficit, congenital infections, severe birth asphyxia (5-min Apgar score <6 or cord blood pH < 7), congenital metabolic or endocrine disease or disorders known to affect growth were excluded from the study. Umbilical cord blood was collected at delivery and cord serum and plasma were separated by centrifugation and stored at −80 °C until analysis. Birth weight and length, gestational age, mode of delivery, five-minute Apgar score and sex were obtained from the hospital birth record. Within 48 h of delivery, research staff measured head and abdominal circumferences and skinfold thickness (subscapular, abdominal, tricep and thigh) of the neonate. Skinfold thickness was measured using a Harpenden Skinfold Caliper (Baty International, West Sussex, UK) by the study coordinator at each site, each of whom was trained by an investigator with extensive experience (DAF).

Maternal age at delivery, gestational weight gain, hemoglobin A1c (if available), results of glucose tolerance screenings, pregnancy history, smoking status and any medications taken during pregnancy were extracted from medical records. Maternal height and pre-pregnancy weight were obtained from medical records if recorded, or by self-report if not, and used to calculate pre-pregnancy body mass index (BMI).

At 1-month of age, infants visited the CMRI Metabolic Research Program facilities at the University of Oklahoma Health Sciences Center for follow-up measurements. Length, weight, head and abdominal circumferences and skinfold thickness were measured and body composition was assessed by dual energy x-ray absorptiometry (DXA; Lunar iDXA, GE-Healthcare, Fairfield, CT). All DXA scans were performed and analyzed by the same person (DAF); suitable scans were obtained in 98.4 % of the infants.

Leptin, high-molecular-weight adiponectin (HMWA), pigment epithelium-derived factor (PEDF) and C-peptide concentrations were measured by ELISA (leptin and HMWA: R&D Systems, Minneapolis, MN; PEDF and C-peptide: EMD Millipore, Billerica, MA) according to the manufacturer’s protocol. All samples were assayed in a single run. Intra-assay variability for leptin, HMWA, PEDF and C-peptide were 2.1 %, 3.2 %, 6.9 %, and 5.8 %, respectively.

Birth analyses include all mother-infant pairs for whom umbilical cord blood, anthropometric measures and maternal records (i.e., the “birth visit”) were available. Thus, data for 79 out of 94 controls and 45 out of 65 DMs were included. Subjects who had cord blood samples available were not different from those who did not in terms of birth anthropometrics or maternal characteristics, except that gestational age was slightly less in those without cord blood samples in the Con-O group (39.0 vs 39.6 weeks, *p* = 0.010; no difference in the DM-O group). Analyses at 1-month include data on all infants for whom both the birth and 1-month visits were completed (53 of 79 Con-O, 36 of 45 DM-O). Participants who completed only the birth visit were not different from those who completed both visits in terms of gestational age, birth anthropometrics or maternal characteristics. The proportions of infants fed formula and/or breastmilk were similar between the groups (% breastmilk/formula/mixed were 24.4/55.6/20.0 in Controls and 20.6/52.9/26.5 in DM). Primary outcomes were infant weight and length; skinfold thickness; percent body fat; fat, lean and fat-free mass; cord serum leptin, HMWA, PEDF and C-peptide.

Group descriptive statistics were expressed as mean ± standard deviation and grouped frequencies. Differences between groups were assessed using the Generalized Linear Model framework. Covariates considered depended on the outcome variable being analyzed and included gestational and maternal age, mode of birth (vaginal or Cesarean), and maternal pre-pregnancy BMI. The covariates included in the model in each case were selected using stepwise methods. While cord blood HMWA and C-peptide values were normally distributed, leptin and PEDF had skewed sampling distributions and were logarithmically transformed to meet the assumptions for linear regression analysis. Correlations were calculated as standardized regression coefficients and adjusted for significant covariates. Data analyses used IBM SPSS Statistics (IBM Corp. Released 2011. IBM SPSS Statistics for Windows, Version 20.0. Armonk, NY: IBM Corp). P-values <0.05 were treated as significant for the purposes of discussion.

**Table 1:** Maternal characteristics

	Control	DM	p-value
N	79	45	--
Age (yr)	24.6 ± 4.4	31.0 ± 6.1	<0.0001
Pre-pregnancy wt (kg)	75.6 ± 18.1	87.4 ± 18.8	0.002
Pre-pregnancy BMI	28.2 ± 6.9	32.7 ± 6.2	0.001
Pregnancy wt gain (kg)	12.6 ± 7.9	9.9 ± 8.3	0.098
Parity	2.2 ± 1.9	3.1 ± 1.8	0.012
HbA1c (%)	5.1 ± 0.3 (*n* = 46)	5.7 ± 0.7 (*n* = 40)	<0.0001

(mean ± SD; unadjusted)

## Results

### Characteristics of mothers

Mothers with DM were 6.4 years older on average and reported increased parity, pre-pregnancy weight and BMI compared to control mothers (Table 1). Fifty-seven percent of control mothers were either overweight (BMI 25.0–29.9 kg/m^2^) or obese (BMI ≥ 30.0 kg/m^2^) by Centers for Disease Control criteria (median BMI = 27.6) whereas 90 % of DM mothers were overweight or obese (median BMI = 32.1). Maternal weight gain during pregnancy for the DM group was about 3 kg less than that of control mothers, but not significant. Parity was higher for DM mothers than for control mothers, possibly reflecting their older average age.

Women with gestational diabetes comprised the majority of the DM group (32 GDM vs. 13 T2DM). There was no difference in cord blood adipokines in offspring of women with T2DM versus GDM so we combined them for subsequent analyses. Dysglycemia generally was well controlled (Table 1), as shown by an average hemoglobin A1c (HbA1c) (available on 40 of the 45 DM mothers) of 5.7 ± 0.7 % (range 4.1–8.1 %). Treatments were as follows: glyburide, 14 mothers; insulin, 18 mothers; metformin, 3 mothers; diet/lifestyle, 7 mothers; not reported, 3 mothers. Additionally, HbA1c values were available for 46 of 79 control mothers, all of whom had normal glucose challenge values, with an average of 5.1 ± 0.3 % (range 4.7–5.9 %; p < 0.0001 versus DM).

### Impact of maternal DM on infant body composition and growth

All infants were born at term (37–42 weeks gestation; Table 2) according to the WHO standard [14]. DM-O were delivered slightly earlier (5 days on average; p < 0.0001) than Con-O, but there was no proportional difference in mode of delivery (vaginal vs. C-section) between groups. On average, DM-O weighed 0.23 kg more than Con-O (3.67 ± 0.59 kg vs. 3.44 ± 0.40, *p* = 0.024), though length and SSF were equivalent between the groups.

At 1-month of age (Table 2), DM-O continued to be about 0.2 kg heavier than Con-O (4.7 ± 0.7 kg vs. 4.5 ± 0.5 kg, *p* = 0.14), but this was no longer a statistically significant difference. There also were no between-group differences in length or SSF and no differences in % fat in the whole body, trunk or limbs as measured by DXA. Furthermore, the change in weight, length and SSF from birth to 1-month did not differ between DM-O and Con-O (Table 2).

### Influence of maternal DM on cord blood adipokine concentrations

Cord blood leptin concentration was increased 2-fold in DM-O compared to Con-O (p <0.0001, Table 2) and unaffected by sex of the infant, mode of delivery, maternal age, pregnancy weight gain or pre-pregnancy BMI. In multivariate analyses accounting for gestational age, SSF, and birth weight (either individually or pairwise), group designation continued to exert an independent effect on cord leptin concentration.

**Table 2:** Infant characteristics

	Control-O	DM-O	p-value
**Birth**			
N	79	45	--
Female/Male	43/36	22/23	0.55
Gestational age (wks)	39.6 ± 0.9	38.9 ± 0.6	<0.0001
Vaginal/C-section Delivery	55/24	31/14	0.93
Weight (kg)	3.4 ± 0.4	3.7 ± 0.6	0.024
Length (cm)	50.1 ± 2.0	49.9 ± 3.0	0.66
Sum of skinfolds (mm)	20.5 ± 4.6	21.6 ± 5.5	0.27
Cord leptin (ng/ml)	9.5 ± 9.9	20.1 ± 14.9	<0.0001
Cord HMWA (ug/ml)	18.2 ± 7.9	16.0 ± 10.4	0.24
Cord PEDF (ug/ml)	3.8 ± 1.4	3.9 ± 1.6	0.78
Cord C-peptide (ng/ml)	0.8 ± 0.5	0.8 ± 0.6	0.91
**1-month**			
N	53	36	
Female/Male	29/24	18/18	0.66
Age at testing (d)	35 ± 5	34 ± 5	0.27
Weight (kg)	4.5 ± 0.5	4.7 ± 0.7	0.14
Length (cm)	53.9 ± 2.4	54.1 ± 2.3	0.73
Sum of skinfolds (mm)	37.4 ± 5.8	38.4 ± 8.2	0.54
Total fat by DXA (%)	24.1 ± 3.1	25.1 ± 2.7	0.12
Trunk fat by DXA (%)	17.0 ± 3.5	17.7 ± 3.1	0.34
Limb fat by DXA (%)	38.1 ± 4.9	39.6 ± 4.0	0.13
Change from birth to 1-month			
delta Weight (kg)	1.0 ± 0.4	1.0 ± 0.5	0.75
delta Sum of skinfolds (mm)	16.2 ± 6.1	16.6 ± 6.9	0.79

(mean ± SD)

**Fig. 1:**
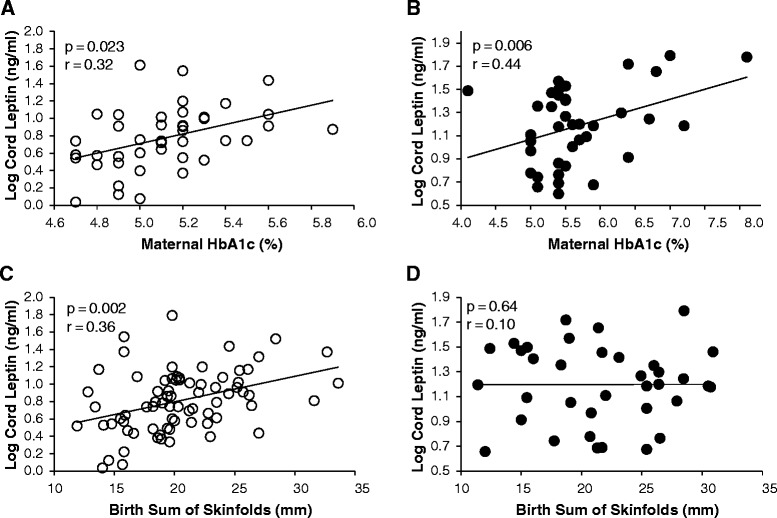
Association of cord blood leptin with maternal hemoglobin A1c. Association of cord blood leptin with maternal hemoglobin A1c (HbA1c) during pregnancy in Con-O (Panel **a**) and DM-O (Panel **b**) and with birth adiposity in Con-O (Panel **c**) and DM-O (Panel **d**). Open circles represent Con-O; closed circles represent DM-O

Only seven of the 40 mothers for whom data were available had an HbA1c greater than 6 %. Significant correlations involving HbA1c remained significant with and without those seven subjects; therefore we chose to include them in the analyses. Maternal HbA1c correlated positively with cord leptin level in the entire cohort (Table 3) and within the separate groups (Fig. 1, Panels a & b). While both groups showed significant positive correlations, there was a significant difference between the groups (*p* = 0.029) with DM-O showing a stronger correlation. Cord PEDF also correlated significantly with maternal HbA1c when the groups were combined (*p* = 0.047).

**Table 3:** Correlations^a^ of cord blood measures with birth, pregnancy and pre-pregnancy measures

	All	p-value	Number	Control-O	p-value	Number	DM-O	p-value	Number	Between-group p-value
**Leptin** ^b^	*DM*			*Gage, parity*			*--*			
Birth weight	**0.23**	**0.009**	121	**0.38**	**0.0007**	79	0.15	0.34	42	**0.025**
Birth SSF	**0.22**	**0.010**	110	**0.36**	**0.002**	74	0.10	0.64	36	**0.025**
Maternal Age	0.00	0.99	120	0.01	0.97	78	0.03	0.86	42	0.79
Maternal Pre-pregnancy BMI	**0.19**	**0.047**	101	0.24	0.06	64	0.05	0.78	37	0.29
Pregnancy weight gain	0.10	0.28	101	0.05	0.70	64	0.12	0.46	37	0.98
Maternal HbA1c	**0.38**	**0.0003**	80	**0.32**	**0.023**	43	**0.44**	**0.006**	37	**0.029**
**HMWA**	*DM*			*--*			*--*			
Birth weight	0.07	0.47	124	0.00	0.99	79	0.18	0.23	45	0.35
Birth SSF	0.10	0.29	113	−0.02	0.86	74	0.28	0.09	39	0.10
Maternal Age	−0.06	0.50	123	0.00	0.99	78	0.01	0.96	45	0.98
Maternal Pre-pregnancy BMI	−0.16	0.12	104	−0.16	0.20	64	−0.11	0.52	40	0.99
Pregnancy weight gain	**0.30**	**0.002**	104	0.13	0.29	64	**0.47**	**0.002**	40	**0.027**
Maternal HbA1c	−0.08	0.48	86	0.12	0.42	46	−0.05	0.78	40	0.44
**PEDF** ^b^	*DM*			*--*			*gender*			
Birth weight	**0.26**	**0.004**	124	**0.28**	**0.014**	79	0.17	0.24	45	0.56
Birth SSF	0.13	0.19	118	0.21	0.07	79	0.17	0.31	39	0.30
Maternal Age	0.20	0.07	123	0.10	0.39	78	0.26	0.07	45	0.54
Maternal Pre-pregnancy BMI	0.16	0.14	104	0.20	0.11	64	−0.02	0.91	40	0.59
Pregnancy weight gain	−0.02	0.82	104	−0.10	0.42	64	0.10	0.54	40	0.36
Maternal HbA1c	**0.24**	**0.047**	86	0.18	0.22	46	0.19	0.21	40	0.64

^a^Correlations shown are standardized correlation coefficients from the regression of cord blood measures on the birth, pregnancy or pre-pregnancy measure with adjustment for significant covariates. Covariates considered were diabetic status (DM), gender, gestational age at birth (Gage), mode of delivery (mode), maternal age and parity. Significant covariates are indicated in italic font

^b^Leptin and PEDF were log transformed for analysis. (*p* ≤ 0.05 in bold)

In contrast with leptin, there was no effect of DM status on cord blood concentrations of HMWA or C-peptide.

### Cord blood adipokine measures and infant body composition and growth

Examination of the relationship between leptin and birth anthropometrics (Table 3) revealed striking group differences. Birth weight and birth SSF were both highly positively correlated with cord leptin in the Con-O group only (Table 3; Fig. 1, Panels c and d). By 1-month of age (Table 4), the relationships between leptin and body size and adiposity were no longer apparent; however, change in weight and SSF from birth to one month was significantly negatively correlated with cord leptin in the entire cohort with no differences in correlation between the groups.

**Table 4:** Correlations^a^ of measures at 1 month with cord blood measures

	All	p-value	Number	Control-O	p-value	Number	DM-O	p-value	Number	Between-group p-value
**Leptin**										
1-month weight	0.04	0.69	86	0.04	0.77	53	−0.11	0.55	33	0.46
1-month SSF	−0.06	0.61	84	0.01	0.95	51	−0.22	0.22	33	0.22
1-month % Total Fat	0.20	0.06	85	0.19	0.19	52	0.07	0.72	33	0.61
Δ Weight (birth to 1-month)	**−0.37**	**0.0005**	86	**−0.35**	**0.009**	53	**−0.49**	**0.004**	33	0.21
Δ SSF (birth to 1-month)	**−0.29**	**0.010**	77	−0.27	0.07	48	**−0.45**	**0.015**	29	0.25
**HMWA**										
1-month weight	**0.26**	**0.004**	89	**0.29**	**0.038**	53	0.18	0.30	36	0.65
1-month SSF	**0.24**	**0.025**	87	0.21	0.15	51	0.27	0.11	36	0.69
% Total fat at 1-month	**0.30**	**0.004**	88	**0.32**	**0.022**	52	0.30	0.07	36	0.49
Δ Weight (birth to 1-month)	**0.22**	**0.044**	89	**0.40**	**0.003**	53	0.06	0.77	36	0.10
Δ SSF (birth to 1-month)	0.21	0.07	80	**0.30**	**0.041**	48	0.11	0.54	32	0.35
**PEDF**										
1-month weight	0.03	0.81	89	0.11	0.49	53	−0.09	0.65	36	0.73
1-month SSF	0.01	0.91	87	0.14	0.32	51	−0.16	0.42	36	0.16
% Total fat at 1-month	0.12	0.23	88	0.07	0.59	52	0.10	0.59	36	0.58
Δ Weight (birth to 1-month)	**−0.31**	**0.003**	89	−0.16	0.26	53	**−0.52**	**0.005**	36	0.15
Δ SSF (birth to 1-month)	−0.19	0.11	80	−0.04	0.78	48	**−0.46**	**0.019**	32	0.20

^a^Correlations shown are standardized correlation coefficients from the regression of the 1-month measures on the cord blood measure with adjustment for significant covariates. Covariates considered were diabetic status (DM), gender, age in days

^b^Leptin and PEDF were log transformed for analysis. (*p* ≤ 0.05 in bold)

For PEDF, birth weight was positively correlated (*p* = 0.0042) with cord concentration (Table 3) and although the effect was only observed in Con-O, no group difference was evident. At 1-month (Table 4), neither body weight nor adiposity (SSF or whole body % fat by DXA) correlated with cord PEDF concentration. Similarly to cord leptin, change in weight and SSF from birth to 1-month was negatively correlated with cord PEDF albeit only significantly in the entire cohort and the DM-O group. There was no significant difference in correlation between the groups.

The relationships between cord HMWA levels and anthropometrics differed from that of leptin and PEDF. Cord HMWA concentration was unrelated to all measures of adiposity and body size at birth, except for a positive correlation with maternal pregnancy weight gain (Table 3). However, at 1-month HMWA was positively correlated with body weight, SSF and whole body % fat in all infants, although these reached significance only in the Con-O group. Furthermore, cord HMWA was positively associated with the change in weight and SSF from birth to 1-month in the Con-O group but not in the DM-O group although the between-group difference was not significant (Table 4).

**Table 5:** Correlations among cord blood hormones

	All	p-value	Number	Control-O	p-value	Number	DM-O	p-value	Number	Between-group p-value
**Leptin**										
HMWA	**−0.34**	**0.0002**	121	**−0.35**	**0.0017**	79	−0.29	0.06	42	0.94
PEDF	**0.45**	**<0.0001**	121	**0.43**	**<0.0001**	79	**0.62**	**<0.0001**	42	**0.009**
C-peptide	−0.01	0.91	121	−0.18	0.11	79	0.18	0.26	42	0.052
**HMWA**										
PEDF	**−0.30**	**0.0009**	124	**−0.27**	**0.014**	79	**−0.32**	**0.031**	45	0.58
C-peptide	0.07	0.46	124	0.06	0.62	79	0.08	0.61	45	0.86

(*p* ≤ 0.05 in bold)

### Cord blood adipokine interrelationships 

**Fig. 2:**
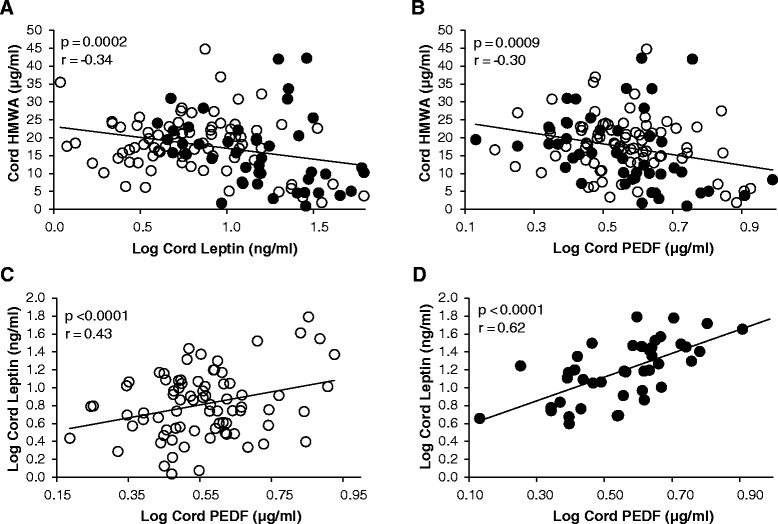
Association of cord blood hormones. Association of cord blood high molecular-weight adiponectin (HMW) with cord blood leptin (Panel **a**) and cord blood PEDF (Panel **b**). Association of cord blood leptin with cord blood pigment epithelium-derived factor (PEDF) in Con-O (Panel **c**) and DM-O (Panel **d**). Open circles represent Con-O; closed circles represent DM-O

Significant correlations were evident among the various measures (Table 5). HMWA correlated negatively with leptin and PEDF in the group as a whole (Fig. 2, Panels a and b). Circulating concentrations of leptin and PEDF were positively correlated (Fig. 2, Panels c and d), with a stronger correlation observed in the DM-O.

## Discussion

We found that maternal DM is associated with fetal hyperleptinemia and disrupts the normal relationship between cord blood leptin and infant body composition. Adiposity at birth (assessed as the sum of skinfold thickness) was unrelated to the concentration of cord leptin in infants of DM mothers, an effect that was independent of birth weight and gestational age. Several previous publications describe increased cord blood leptin levels in offspring of women with T2DM, T1DM, or GDM. While there is general agreement that maternal DM raises cord blood leptin level (save Pirc et al [15] and Lea et al [16], who report no such effect), there is controversy about underlying mechanisms. Indeed, Okereke et al [17] found that, while cord leptin levels were higher in infants of gestational DM than non-diabetic mothers, adjustment for infant fat mass abrogated the relationship, leading to the conclusion that the effect of maternal DM is due to increased fetal adiposity. This contrasts with our findings, in which birth adiposity is unrelated to cord leptin in infants of DM mothers. The reason for this discrepancy is not entirely clear but may relate to differing methods for determining fat mass and/or different study populations. In a study of mostly T1DM women, Persson et al [18] reported increased cord leptin in DM offspring with no correlation with birth weight in the infants of mothers with DM. Similarly, Gross and colleagues [19] found that the presence of maternal DM correlated with cord blood leptin, independent of birth weight. The latter two studies, although lacking measures of adiposity, support our conclusion that an increase in fetal fat mass does not explain the effect of maternal DM on cord leptin concentration.

Our data suggest that maternal glycemia is a dominant determinant of fetal leptin concentration. We found a positive relationship between cord leptin and maternal HbA1c in offspring of diabetic mothers, in agreement with two prior studies [20, 21]. Additionally, we observed a correlation between cord leptin and maternal HbA1c in the controls. Neither cited study examined the relationship with fetal leptin and maternal glycemia in uncomplicated pregnancies. In fact, the results of our study are consistent with data from the HAPO study of more than 20,000 non-diabetic pregnancies, which show that modest increments in maternal glycemia are associated with increases in birth weight [22]. Thus, the degree of glucose exposure during normal pregnancy plausibly underlies the relationship of adiposity and leptin.

Another important finding in our study is the relationship between cord blood adiponectin and adiposity at 1-month of age. Although the broader significance of this finding is unclear at this time, adiponectin levels remain elevated (relative to adult values) for the first six months of life [23], supporting the notion that the adipokine is involved in the expansion of fat mass following birth. Several previous studies, like ours, find no effect of maternal DM on cord blood adiponectin concentration [24, 25], whereas others report a modest decrease [26]. Similarly, some, but not all, studies report positive correlations with birth anthropometrics [24, 27, 28]. The reasons for these differences are unclear, but may reflect differing assay methodologies and whether total or high molecular weight adiponectin was measured.

The role of PEDF during human development and childhood is largely unexplored. We found a modest relationship between birth weight and cord blood PEDF overall with no differences between infants born to mothers with DM versus controls. However, there were striking correlations with cord blood levels of both HMWA (negative) and leptin (positive). The strongest relationship was between leptin and PEDF in the offspring of DM mothers, wherein 38 % of the variation in leptin concentration was explained by PEDF level. Although the basis for this relationship is unclear, it could be reflect the need for the angiogenic properties of leptin to be balanced by the anti-angiogenic effects of PEDF in the placenta, a putative source of cord blood leptin and PEDF [10, 29]. PEDF has been shown to inhibit leptin induced angiogenesis *in vitro* [30]. Furthermore, Böhm et al have found a common genetic variation in the PEDF gene among individuals at risk for T2DM that is associated with increases in circulating concentrations of both PEDF and leptin [31], providing an additional link between these cytokines as well a potential explanation for the relationship.

The strengths of this study are the careful phenotyping of the infants at birth, the detailed measures of growth and body composition over the first month of life, and the new insights concerning potential determinants of cord leptin, PEDF, and the connection between HMWA and the acquisition of adipose tissue. The conclusions, however, are based on correlations and thus cause and effect relationships remain to be determined. In addition, we do not know whether the observations concerning PEDF and leptin reflect changes in expression by placenta or other fetal tissues such as liver or adipose depots. A potential limitation is the attrition between the birth and 1-month visits (due largely to inability to reach the mothers by phone, letter or email). However, there were no obvious distinctions between subjects who completed only the birth visit and those who completed both visits, suggesting this had minimal impact on our findings. Treatment modalities varied amongst the DM mothers, with glyburide and insulin most commonly prescribed. Though neither birth weight [32] nor neonatal adiposity [33] appear to be affected differently by glyburide versus insulin, we cannot rule out the possibility that other outcomes may be impacted by treatment. Finally, other metabolic changes that are coincident with maternal DM (e.g. hyperlipidemia) were not assessed. Future studies will be required to dissect out which of the effects reflect changes induced by maternal metabolic aberration and which may be due to specific genetic variations passed on to offspring and to what extent they relate to long term health of the offspring.

## Conclusions

Cord blood leptin at term is increased by maternal diabetes independently of neonatal adiposity and is directly related to maternal hemoglobin A1c percent in offspring of diabetic and non-diabetic women. These data provide further evidence that infants born to mothers with near-optimal management of diabetes still show the effects of the condition and that the level of maternal glycemia has a major influence on leptin secretion. In contrast, PEDF concentration is unaffected by dysglycemia, but correlates with birth weight and leptin, suggesting a novel role(s) for this understudied cytokine in fetal growth control and placental function. Lastly, higher levels of cord blood HMWA were associated with increased infant growth and adiposity at 1 month, supporting a role for HMWA in the acquisition of fat mass during infancy.

## Abbreviations

Con-O: Offspring of control-group mothers
DM: Diabetes mellitus
DM-O: Offspring of diabetic mothers
DXA: Dual energy x-ray absorptiometry
HbA1c: Hemoglobin A1c
HMWA: High molecular-weight adiponectin
PEDF: Pigment epithelium-derived factor
SSF: Sum of skinfolds
